# Daily estimation of the severity of organ dysfunctions in critically ill children by using the PELOD-2 score

**DOI:** 10.1186/s13054-015-1054-y

**Published:** 2015-09-15

**Authors:** Stéphane Leteurtre, Alain Duhamel, Valérie Deken, Jacques Lacroix, Francis Leclerc, on behalf of the Groupe Francophone de Réanimation et Urgences Pédiatriques (GFRUP)

**Affiliations:** Pediatric Intensive Care Unit, Jeanne de Flandre University Hospital, 2 avenue Eugène Avinée, 59037 Lille, Cedex France; EA 2694, Public Health: Epidemiology and Quality of Care, University of Lille 2, Lille, France; Department of Biostatistics, University of Medicine, Lille, France; Pediatric Intensive Care Unit, Sainte-Justine Hospital, Université de Montréal, Montréal, Canada

## Abstract

**Introduction:**

Daily or serial evaluation of multiple organ dysfunction syndrome (MODS) scores may provide useful information. We aimed to validate the daily (d) PELOD-2 score using the set of seven days proposed with the previous version of the score.

**Methods:**

In all consecutive patients admitted to nine pediatric intensive care units (PICUs) we prospectively measured the dPELOD-2 score at day 1, 2, 5, 8, 12, 16, and 18. PICU mortality was used as the outcome dependent variable. The discriminant power of the dPELOD-2 scores was estimated using the area under the ROC curve and the calibration using the Hosmer-Lemeshow chi-square test. We used a logistic regression to investigate the relationship between the dPELOD-2 scores and outcome, and between the change in PELOD-2 score from day1 and outcome.

**Results:**

We included 3669 patients (median age 15.5 months, mortality rate 6.1 %, median length of PICU stay 3 days). Median dPELOD-2 scores were significantly higher in nonsurvivors than in survivors (p < 0.0001). The dPELOD-2 score was available at least at day 2 in 2057 patients: among the 796 patients without MODS on day1, 186 (23.3 %) acquired the syndrome during their PICU stay (mortality 4.9 % vs. 0.3 % among the 610 who did not; p < 0.0001). Among the1261 patients with MODS on day1, the syndrome worsened in 157 (12.4 %) and remained unchanged or improved in 1104 (87.6 %) (mortality 22.9 % vs. 6.6 %; p < 0.0001). The AUC of the dPELOD-2 scores ranged from 0.75 (95 % CI: 0.67-0.83) to 0.89 (95 % CI: 0.86-0.91). The calibration was good with a chi-square test between 13.5 (p = 0.06) and 0.9 (p = 0.99). The PELOD-2 score on day1 was a significant prognostic factor; the serial evaluation of the change in the dPELOD-2 score from day1, adjusted for baseline value, demonstrated a significant odds ratio of death for each of the 7 days.

**Conclusion:**

This study suggests that the progression of the severity of organ dysfunctions can be evaluated by measuring the dPELOD-2 score during a set of 7 days in PICU, providing useful information on outcome in critically ill children. Its external validation would be useful.

## Introduction

In the intensive care unit (ICU), almost all adult and pediatric patients present some organ dysfunction [1–5] and mortality rates increase with the number of organ dysfunctions [5–8]. Organ dysfunction scores were developed in critically ill adults and children to describe and quantify the severity of organ dysfunctions throughout the ICU stay. These scores are frequently used as an outcome variable in clinical trials [9–11]. In 2003 we developed and validated a multiple organ dysfunction syndrome (MODS) score for critically ill children: the pediatric logistic organ dysfunction (PELOD) score using the most abnormal value of each variable during the entire pediatric ICU (PICU) stay [12]. In 2010, considering that measurements repeated daily may provide more useful information, we identified a set of 7 days as the optimal period for measurement of the daily PELOD score [13]. In 2013, using a larger and more recent database, we developed and validated the PELOD-2 score, which, contrary to the first version, uses a continuous scale [14]. The objective of this study was to validate the daily PELOD-2 (dPELOD-2) score using the set of 7 days proposed with the previous version of the PELOD score [13].

## Materials and methods

All consecutive patients admitted between June 2006 and October 2007, to nine multidisciplinary, tertiary-care PICUs of university-affiliated hospitals (eight French and oneBelgian, all member of the Groupe Francophone de Réanimation et Urgences Pédiatriques-GFRUP) were prospectively included. Exclusion criteria were: age 18 years or older; premature at entry into PICU; pregnancy; total length of stay in PICU less than 4 h; admission in a state of continuous cardiopulmonary resuscitation without achieving stable vital signs for at least 2 h; transfer from another PICU; and admissions for scheduled procedures normally performed in other hospital locations. The study and its database were declared safe and were approved by the French authorities (Commission Nationale de l’Informatique et des Libertés) on 7 February 2007. The study design was approved by the ethics committee of the Société de Réanimation de Langue Française on 27 April 2007 for all the participating hospitals. The requirement for consent was waived because the study was strictly observational.

We collected baseline characteristics and calculated dPELOD-2 score at days 1, 2, 5, 8, 12, 16 and 18 in PICU. The PICU day 1 started from admission time to H24, and so on. Length of PICU stay was defined as the difference between admission day and discharge day plus 1. For each variable, the most abnormal value each day was used in calculating the dPELOD-2 score [12, 14]. Variables were measured only if requested by the attending physician (i.e., if justified by clinical status of patient). Every day, if a variable was not measured, we assumed that it was identical to the previous measurement (i.e., the physician considered that the value of the variable did not change) or normal (i.e., the physician considered that the value of the variable was normal) [12, 14]. Organ dysfunction was defined as a PELOD-2 score >0 for a given organ, and MODS as the simultaneous presence of two or more organ dysfunctions The PICU discharge status (death/survival) was used as the outcome dependent variable.

### Statistical analysis

All statistical analyses were performed with SAS software (SAS institute Inc., Cary, NC, USA). A *p* value <0.05 was considered statistically significant. Results are expressed as frequencies and percentages for categorical variables and as median and interquartile range (IQR) for quantitative variables. The comparisons between the two groups of outcomes and the quantitative variables were performed using the Wilcoxon signed rank test. The relationships between the outcome and the categorical variables were analyzed using the chi-square test or Fischer’s exact probability test.

The discriminant power of the dPELOD-2 scores was estimated using the area under the receiver-operating characteristics curve (AUC) (with 95 % confidence interval) and the calibration was assessed using the Hosmer-Lemeshow chi-square test. We used logistic regression to investigate the relationship (1) between the dPELOD-2 scores and outcome, and (2) between the change in PELOD-2 score from day 1 and outcome.

## Results

The study included 3,669 patients, two patients of the original database [14] with incomplete data being excluded. Characteristics of the population are reported in Table 1. The mortality rate was 6.1 % (222 deaths). Administrative median length of stay in PICU was 3 days [2–6] in survivors and 4 days  [2–12] in non survivors (*p* = 0.055).

**Table 1:** Population characteristics

Variable	Value
Total number of patients	3,669
Baseline characteristics	
Gender, male, n (%)	2096 (57.1)
Age, months, median (IQR)	15.5 (2.2; 70.7)
0 to <1 months, n (%)	627 (17.1)
1 to 11 months, n (%)	1067 (29.1)
12 to 23 months, n (%)	398 (10.9)
24 to 59 months, n (%)	559 (15.2)
60 to 143 months, n (%)	562 (15.3)
≥144 months, n (%)	456 (12.4)
Recovery post procedure, n (%)^a^	955 (26.0)
PIM2 score, predicted death rate in %, median (IQR)	1.43 (0.78; 4.33)
Primary reason for PICU admission, n (%)	
Respiratory	1663 (45.3)
Neurological	662 (18.0)
Cardiovascular	672 (18.3)
Hepatic	40 (1.1)
Genitourinary	96 (2.6)
Gastrointestinal	205 (5.6)
Endocrine	57 (1.6)
Musculoskeletal	45 (1.2)
Hematological	45 (1.2)
Miscellaneous/undetermined	99 (2.7)
Mixed	85 (2.3)
Cause of primary diseases at entry, n (%)	
Infection	862 (23.5)
Trauma	324 (8.8)
Congenital disease	1123 (30.6)
Drug poisoning	72 (2.0)
Cancer	120 (3.3)
Diabetes	41 (1.1)
Allergic/immunologic diseases	55 (1.5)
Miscellaneous/undetermined	1072 (29.2)
Elective PICU admission, n (%)^a^	970 (26.4)
Outcomes	
Mechanical ventilation, n (%)	1926 (52.5)
Length of ICU stay, days, median (IQR)	3 (2; 6)
Mortality, n (%)	222 (6.1)

^a^According to pediatric index of mortality (PIM2) instructions. *IQR* interquartile range, *PICU* pediatric intensive care unit

**Fig. 1:**
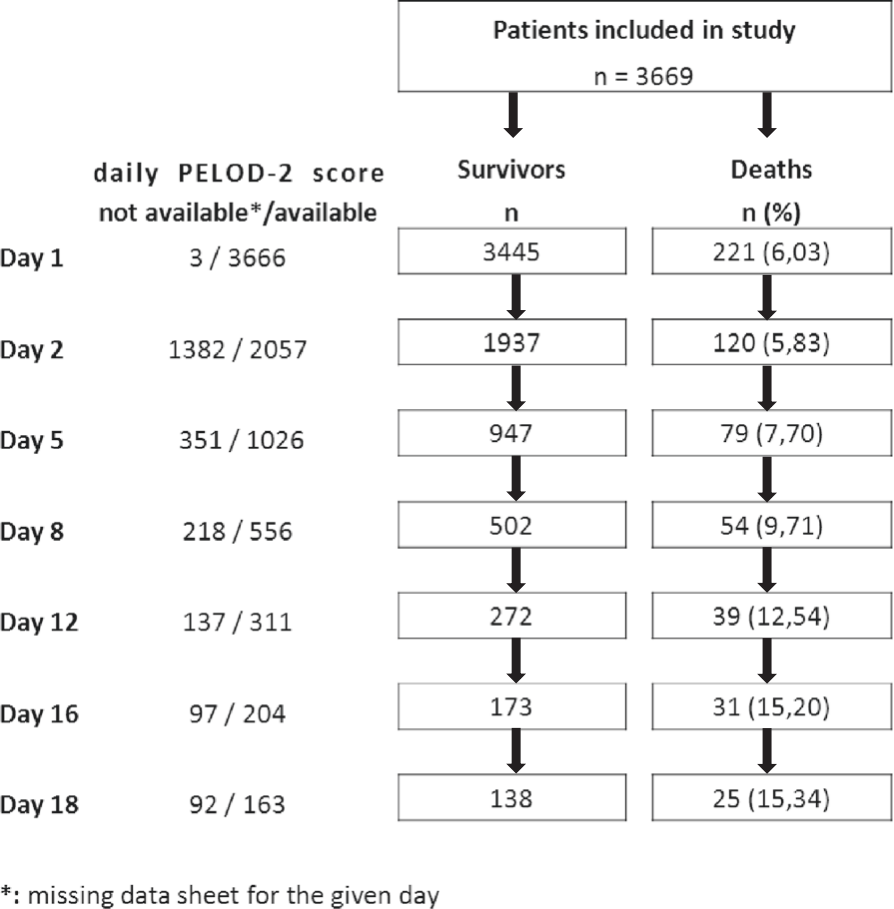
Selection of critically ill children for daily pediatric logistic organ dysfunction (*PELOD-2*) score measurements

Daily PELOD-2 score was measured on 7,983 days (Fig. 1). MODS was present on day 1 in 2,024 of the patients in the whole population (55.2 %). The dPELOD-2 score was available at least at day 2 in 2,057 patients (Fig. 1) allowing the identification of new and progressive MODS: among the 796 patients without MODS (one or no organ dysfunction) on day 1, 186 (23.3 %) acquired the syndrome during their PICU stay (new MODS); the mortality rate was 4.9 % among these children, as compared with 0.3 % among the 610 who did not acquire the syndrome during their stay (*p* <0.0001). Among the 1,261 patients with MODS on day 1, the syndrome worsened during PICU stay in 157 (12.4 %) (progressive MODS) and remained unchanged or improved in 1,104 (87.6 %); the mortality rate was 22.9 % among those in whom it worsened and 6.6 % among the other children; *p* <0.0001). New or progressive MODS was reported in 343 patients (9.3 % of the whole population).

The PELOD-2 score on day 1 was a significant prognostic factor; the serial evaluation of the change in the dPELOD-2 score from day 1, adjusted for baseline value, demonstrated a significant odds ratio of death for each of the 7 days (Table 2). Median (IQR) values of dPELOD-2 scores in survivors and non-survivors are given in Table 3. The median maximum PELOD-2 score was 5 (2–7) and differed between survivors and non-survivors (5 (2–7) and 15 (10–20), *p* <0.0001). The AUC of the dPELOD-2 scores at days 1, 2, 5, 8, 12, 16 and 18 in PICU, ranged from 0.75 (95 % CI 0.67, 0.83) to 0.89 (95 % CI 0.86, 0.91) indicating moderate to good discrimination (Table 3). The calibration assessed by the Hosmer-Lemeshow chi-square test ranged from a chi squared statistic of 13.5 (*p* = 0.06) to 0.9 (*p* = 0.99), indicating good calibration (Table 3). For each of the 7 days under evaluation, mortality significantly increased with the number of organ dysfunctions (Table 4).

**Table 2:** Serial evaluation of the change in the daily PELOD-2 score from day 1, adjusted for baseline value (PELOD-2 score on day 1)

Variable	Odds ratio	95 % CI	*P* value
PELOD-2 score on day 1	1.51	1.44, 1.57	<.0001
Change in PELOD-2 score			
Day 1 to day 2	1.30	1.21, 1.41	<.0001
Day 1 to day 5	1.36	1.25, 1.48	<.0001
Day 1 to day 8	1.37	1.23, 1.53	<.0001
Day 1 to day 12	1.30	1.14, 1.49	<.0001
Day 1 to day 16	1.45	1.23, 1.71	<.0001
Day 1 to day 18	1.44	1.21, 1.72	<.0001

Odds ratio (OR) for death are given with 95 % CI. The cumulative OR of death was calculated as follows: (OR of pediatric logistic organ dysfunction (PELOD) score on day1) × (OR for change in score from day1 to specified day). For example, for a child whose score is 10 on day1 and 5 on day12, the change in score is −5; the OR for death would be 16.60 = ((1.51^10^) × (1.3^−5^)). For a child whose score is 4 on day 1 and 10 on day 8, the change in score is 6; in this instance, the OR for death would be 34.37 = ((1.51^4^ × 1,37^6^))

**Table 3:** Daily PELOD-2 scores among critically ill children: discrimination and calibration

Day	PELOD-2 score, survivors, median (IQR)	PELOD-2 score, non-survivors, median (IQR)	*P* value	Discrimination AUC (CI 95 %)	Calibration^a^ chi-square (*p* value)
1	4 (2–6)	12 (8–18)	0.0001	0.89 (0.86, 0.91)	7.7 (0.47)
2	3 (2–5)	8 (5–15)	0.0001	0.83 (0.78, 0.87)	13.5 (0.06)
5	3 (2–5)	7 (5–10)	0.0001	0.80 (0.75, 0.85)	2.3 (0.89)
8	3 (2–5)	7 (5–9)	0.0001	0.80 (0.73, 0.86)	0.9 (0.99)
12	3 (2–5)	6 (5–8)	0.0001	0.75 (0.67, 0.83)	3.0 (0.80)
16	3 (2–5)	6 (4–8)	0.0001	0.78 (0.69, 0.87)	5.2 (0.51)
18	3 (2–5)	6 (5–8)	0.0001	0.80 (0.72, 0.89)	7.6 (0.27)

^a^Hosmer-Lemeshow goodness-of-fit test. *PELOD* pediatric logistic organ dysfunction, *IQR* interquartile range, *AUC* area under the receiver operating characteristic curve

**Table 4:** Daily mortality (in percentage) related to organ dysfunction number

OD number	0	1	2	3	4	5	*P* value^a^
Day 1	0.7	0.8	3.0	9.6	38.0	70.2	<0.0001
Day 2	1.1	1.7	4.3	10.9	36.1	70.6	<0.0001
Day 5	0.6	3.2	8.2	20.1	28.6	50.0	<0.0001
Day 8	1.1	4.2	10.6	24.1	35.0	0	<0.0001
Day 12	3.7	4.9	15.7	27.6	25.0	0	<0.0001
Day 16	4	6.1	18.7	34.6	57.1	NA	<0.0001
Day 18	0	6.9	18.4	38.1	44.4	NA	<0.0001

^a^Mann–Whitney test. *OD* organ dysfunction, *NA* not applicable

## Discussion

In this study, we report that the dPELOD-2 scores calculated on the 7 days previously identified, had good discrimination and calibration. This study confirms that the progression of the severity of organ dysfunctions can be evaluated by measuring the dPELOD-2 scores during a specified set of days in PICU (admission and days 2, 5, 8, 12, 16 and 18). Our data not only showed that the PELOD-2 score on day 1 was a significant prognostic factor, but also that mortality was significantly higher in children in whom MODS worsened after day 1 as compared with those in whom MODS remained unchanged or improved. Thus, it makes sense to collect data daily on the severity of MODS in order to take into account this time factor [15, 16]. It has been suggested that MODS scores are effective in quantifying the severity of each organ failure during the first ICU days, in adults [17–19] as well as in children [20]. Furthermore, there is increasing evidence that the duration and progression of MODS influence outcome, indicating that MODS is a dynamic process [15, 20–28].

One can question if it is useful to monitor dPELOD-2 scores during a predetermined set days. Many studies have demonstrated that daily organ dysfunction monitoring can be a useful measurement to estimate the response to therapy in a group of patients [29–32]. The model that we proposed in this study, which includes 7 days distributed over the PICU stay, may represent the best balance between the workload of assessing daily scores and the optimal association with prognosis throughout the PICU stay. There have indeed been a number of studies in critically ill adults that support such an approach [18, 21, 33]. Moreover, in studies of long-stay ICU patients, severity scores at admission fail to predict mortality [18, 34]. The late events cannot be predicted with admission or first-day predictive scores, and this suggest that for patients with prolonged ICU stay, the calculation of scores on later days, for example on days 8, 12, 16 and 18, may be useful.

What could be the practical applications of daily MODS scores? Some pediatric intensivists consider that an effective assessment of the severity of MODS, like the PELOD-2 score, is needed to correctly describe the clinical course in critically ill children [10]. The MODS score can be used as an outcome measure in trials conducted in the ICU [35, 36] and PICU [11, 30, 37, 38]. A recent study reported that delayed or inappropriate antimicrobial administration beyond 3 h from recognition of sepsis is an independent risk factor for prolonged organ failure and mortality [38]. Our study showed that the progression of the dPELOD-2 score added information to the PELOD-2 score for the whole PICU stay. All these data support the concept that the PELOD-2 score and its progression in the PICU can be outcome measures of interest in quality assessment activities and in clinical trials. New approaches, such as dynamic Bayesian networks, using the sequential organ failure assessment (SOFA) score, suggest that a sequence of organ dysfunctions can be predicted, which allows physicians to anticipate the development of MODS and help them undertake therapeutic decisions [39].

Strengths of this study include that it was prospective, and it was conducted in nine PICUs across two countries. It included a large number of critically ill children (3,669) and a huge number of days in PICU (more than 7,900 days). Indeed, we have taken into account the dynamics of organ dysfunctions throughout the entire PICU stay.

This study is not without limitations. First, the dataset was collected 8 years ago and the case mix and mortality rate may have changed over this time period. Second, it was conducted in only two European countries (France and Belgium); our population is different from the US and UK populations [14]. Last, the number of deaths was quite low after 7 days in the PICU (there were 54 deaths in 557 patients with a length of stay in the PICU longer than 7 days), but higher than that in our previous study (33 deaths in 338 patients) [13]. Therefore, a large prospective study that is not country-specific would be useful to evaluate external validity of the PELOD-2 score; it should include more critically ill children with a long-term PICU stay and consider two groups of patients, those with a short PICU stay (<7 days) and those with a long PICU stay (≥7 days), in order to collect a sufficient number of deaths in each group [40]. Interestingly, a recent Portuguese study including 556 critically ill children admitted to PICU from January 2011 to December 2012 reported good discrimination (AUC 0.94) and calibration (after recalibration) of the PELOD-2 score [41].

## Conclusion

This study shows that the progression of the severity of organ dysfunctions can be evaluated by measuring the dPELOD-2 score during a specified set of 7 days in the PICU. The serial evaluation of the change in the dPELOD-2 score from day 1, adjusted for the baseline value, demonstrated a significant odds ratio of death for each of the 7 days. The daily PELOD-2 score could be a helpful tool to stratify critically ill children enrolled in clinical trials, to describe their clinical course, to estimate therapeutic responses and to describe outcomes. It could also be used for epidemiologic and administrative purposes. External validation of the PELOD-2 score needs additional studies including more patients with a PICU stay ≥7 days.

## Key messages

- Progression of the severity of MODS can be evaluated by measuring the dPELOD-2 score during a specified period of 7 days in the PICU
- Daily PELOD-2 scores calculated on these days had good discrimination and calibration when used to predict short-term mortality
- In children in the PICU, the PELOD-2 score on day 1 is a significant prognostic factor
- New or progressive MODS during the PICU stay is associated with an increased risk of mortality
- The serial evaluation of the change in the dPELOD-2 score from day 1, adjusted for baseline value, demonstrated a significant odds ratio of death for each day

## Abbreviations

AUC: area under the curve
dPELOD: daily pediatric logistic organ dysfunction
GFRUP: Groupe Francophone de Réanimation et Urgences Pédiatriques
ICU: Intensive care unit
IQR: interquartile range
MODS: multiple organ dysfunction syndrome
PELOD: pediatric logistic organ dysfunction
PICU: pediatric intensive care unit
